# A Summary of Online Enquiries Submitted to Anti-doping Hotline 2005–2018

**DOI:** 10.3389/frph.2021.787954

**Published:** 2021-12-15

**Authors:** Lena Ekström, Susanne Broström, Marja-Liisa Dahl, Annica Börjesson

**Affiliations:** ^1^Division of Clinical Pharmacology, Department of Laboratory Medicine, Karolinska Institute, Karolinska University Hospital, Stockholm, Sweden; ^2^Department of Clinical Pharmacology, Karolinska University Hospital, Stockholm, Sweden

**Keywords:** anabolic androgenic steroids, AAS, doping, side effects, online enquiries, Anti-Doping Hotline, SARM

## Abstract

Anabolic Androgenic Steroid (AAS) abuse in the society is considered a health problem and has been associated with cardiovascular toxicity, endocrine disruption, as well as psychiatric symptoms such as aggression and cognitive dysfunction. Men and women abusing AAS, as well as persons in close relationship to AAS abusers, may encounter concerns. Subsequently, the Anti-Doping Hotline was formed 1993 to answers questions about doping in the society. Here we have reviewed 7,123 enquiries posted on the Anti-Doping Hotline website between 2005 and 2018 to see what type of questions were raised. Most questions (*n* = 2,924) involved AAS, 60% from abusers themselves, and 17% from a person close to an AAS abusers. Only 2.3% of the questions concerned AAS abusing women. Of the AAS specific questions most were from persons who sought personal advice regarding risks and side effects. Notably, the AAS abusers themselves were concerned about somatic side effects (e.g., gynecomastia) and problems related to the AAS injection. The persons in close relationship to an AAS abusers on the other hand, expressed concerns about psychiatric changes including mood swings and aggressivity. In addition to AAS, 26 and 13% of the questions involved dietary supplements and other doping substances, respectively. A gradual decrease of questions regarding ephedrine was noted, whereas the numbers of SARMs related questions increased during this time. Our results show that there is a continuous need to provide medical, nursing, and social support and counseling to AAS abusers and their next of kin.

## Introduction

Anabolic androgenic steroids (AAS) are a family of hormones that comprises testosterone and it's synthetic derivatives that are abused for their muscle growth and performance enhancing effects (1). AAS are forbidden in elite sport and every year, the World Anti-Doping Agency (WADA) publishes a list of prohibited substances comprising over 60 AAS and their metabolites. In many countries, AAS are also forbidden outside the WADA community i.e., they are illegal to possess and sell, and in some countries even to administer. AAS have been associated with many somatic and psychiatric adverse effects, and 88–99% of AAS abusers report subjective adverse effects (2, 3). AAS abuse can be considered a health problem worldwide (4), that may increase with the aging of AAS abusing populations (5).

Consequently, there is a need for a public counseling support where questions and concerns can be raised about doping in general and AAS in particular, by people not engaged in elite sports. Such a service should be provided by medical expertise with in-depth knowledge about AAS. In some countries telephone and internet counseling to non-elite athletes is provided by the national Anti-doping organizations (6), whereas in Sweden, this service to the society is available since 1993 at the Anti-Doping Hotline (ADHL), within the healthcare. In 2003, a summary of the questions raised in the telephone calls received between 1993 and 2000 was published. It was shown that most calls were from male AAS abusers encountering adverse side effects, the most commonly reported being aggressiveness, depression, acne and anxiety (7).

Since 2005 it is possible to submit enquiries to ADHL online, on it's webpage. The aim of this study was to categorize and analyze the enquiries posted on our homepage in relation to type of question, year, gender, and the AAS substances stated, to obtain more knowledge on what type of anti-doping support individuals outside elite sports look for. The results may be of interest for future information and counseling services to the public and recreational athletes.

## Materials and Methods

The ADHL provides nation-wide information and education about doping agents and performs research in the field. ADHL was conceived from our Anti-Doping Laboratory in 1993 as an independent service as a consequence of the laboratory receiving many calls from the public about doping and its medical consequences (7). The service is managed by trained nurses and clinical pharmacologists at the Department of Clinical Pharmacology, Karolinska University Hospital, Stockholm, Sweden. Since 2005 it has been possible to submit enquiries not only by phone but also online *via* the ADHL homepage www.dopingjouren.se.

This study included all online enquiries submitted to ADHL between January 2005 and December 2018. On the homepage the questions are submitted as written free text with no additional information required except for the questioner's e-mail address to which the answer will be sent. The questions were upon arrival grouped by a nurse into one of the following categories: (1) AAS, (2) dietary supplements (3) other doping substances, (4) sports related questions, (5) questions from students/pupils, (6) narcotics, and (7) other not specified questions. All the questions were stored in a database and the questioners' e-mail addresses were erased before the data were compiled in this study to protect the identity of those making enquiries. Consequently, there were large variations in content and composition of the questions. Herein, the questions were further assessed by the investigators and divided into subgroups based on who the questioner was and the content of the question, as presented in the results.

To investigate the correlations between the numbers of questions posted at ADHL and positive findings of specified AAS substances at our Abuse Laboratory [previously reported in Widing et al. (8)]. Pearson test was performed (Graph Prism 8.0).

## Results

### Summary of All Enquiries

**Figure 1:**
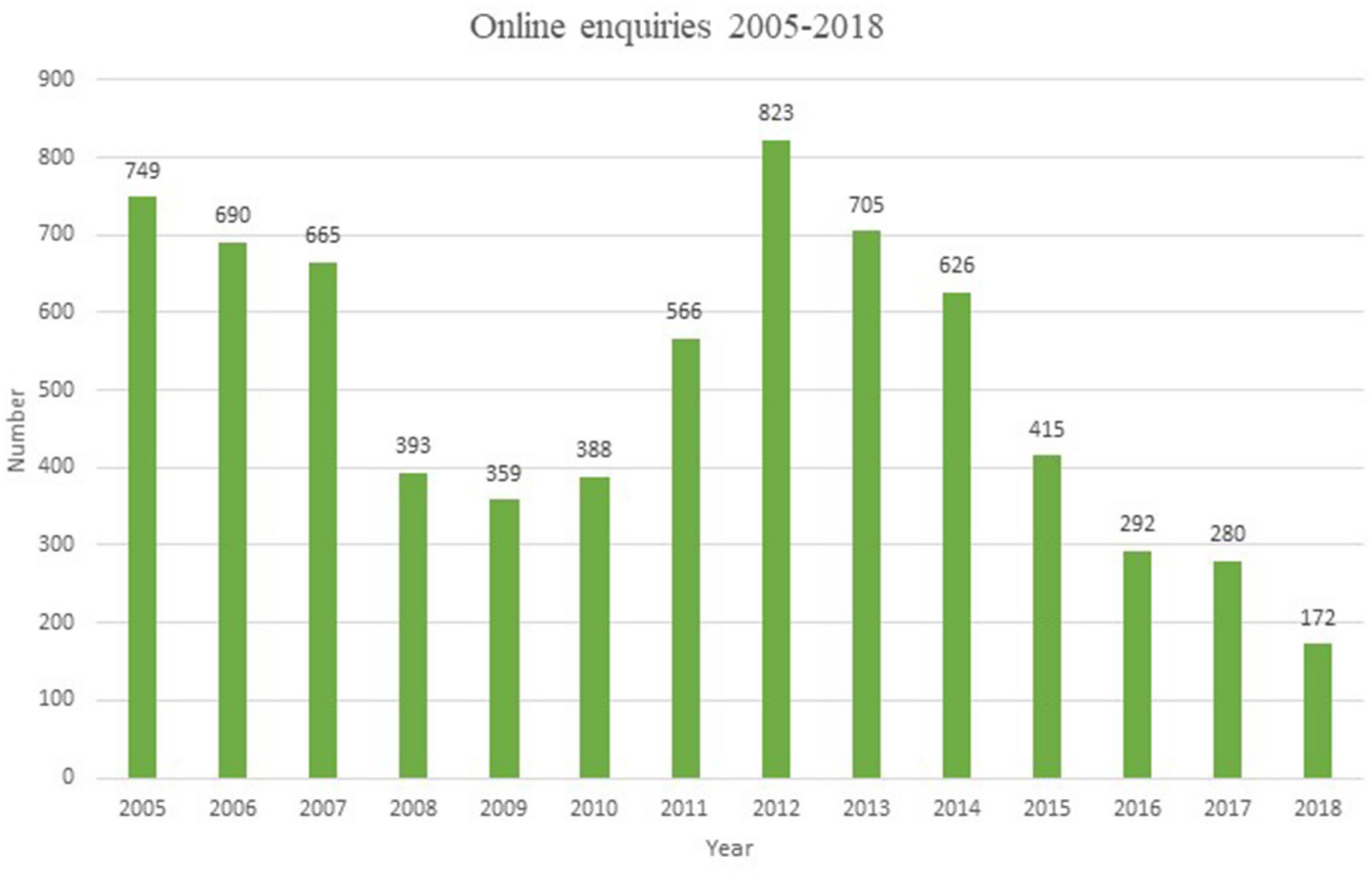
Total number of enquiries between 2005 and 2018.

In total 7,123 enquiries were submitted to the ADHL homepage between 2005 and 2018. The number of enquiries per year varied between 172 and 823 (mean 509 questions per year) (Figure 1).

The majority of the incoming questions were about AAS (Table 1). In addition, questions were asked about dietary supplements and other doping substances. There were also questions related to competitive sports (often concerning to therapeutic drug use and the prohibited list) and questions from students. Only a few questions concerned narcotics.

**Table 1:** Categories of the incoming questions at the website.

**Online enquiries**	**Number (%)**
Anabolic androgenic steroids (AAS)	2,924 (41)
Dietary supplements	1,843 (25.9)
Other doping substances	893 (12.6)
Sports related	451 (6.3)
From students	402 (5.6)
Narcotics	60 (0.8)
Not specified	550 (7.7)

### AAS Related Enquiries

There were 2,924 AAS related questions that mainly came from AAS abusers themselves (60%), from a person in close relationship with an AAS abuser (17%), or from professionals in the healthcare, police etc. (2%). In 21% of the questions, the data did not allow categorization of the questioner.

Most of the questions came from or regarded AAS abusing men. Only 52 (2.3%) of the AAS related questions concerned women. Most of these were from the AAS using women themselves (75%), and the rest from their next of kin (25%). The number of questions from women varied between 1 and 8 per year.

**Figure 2:**
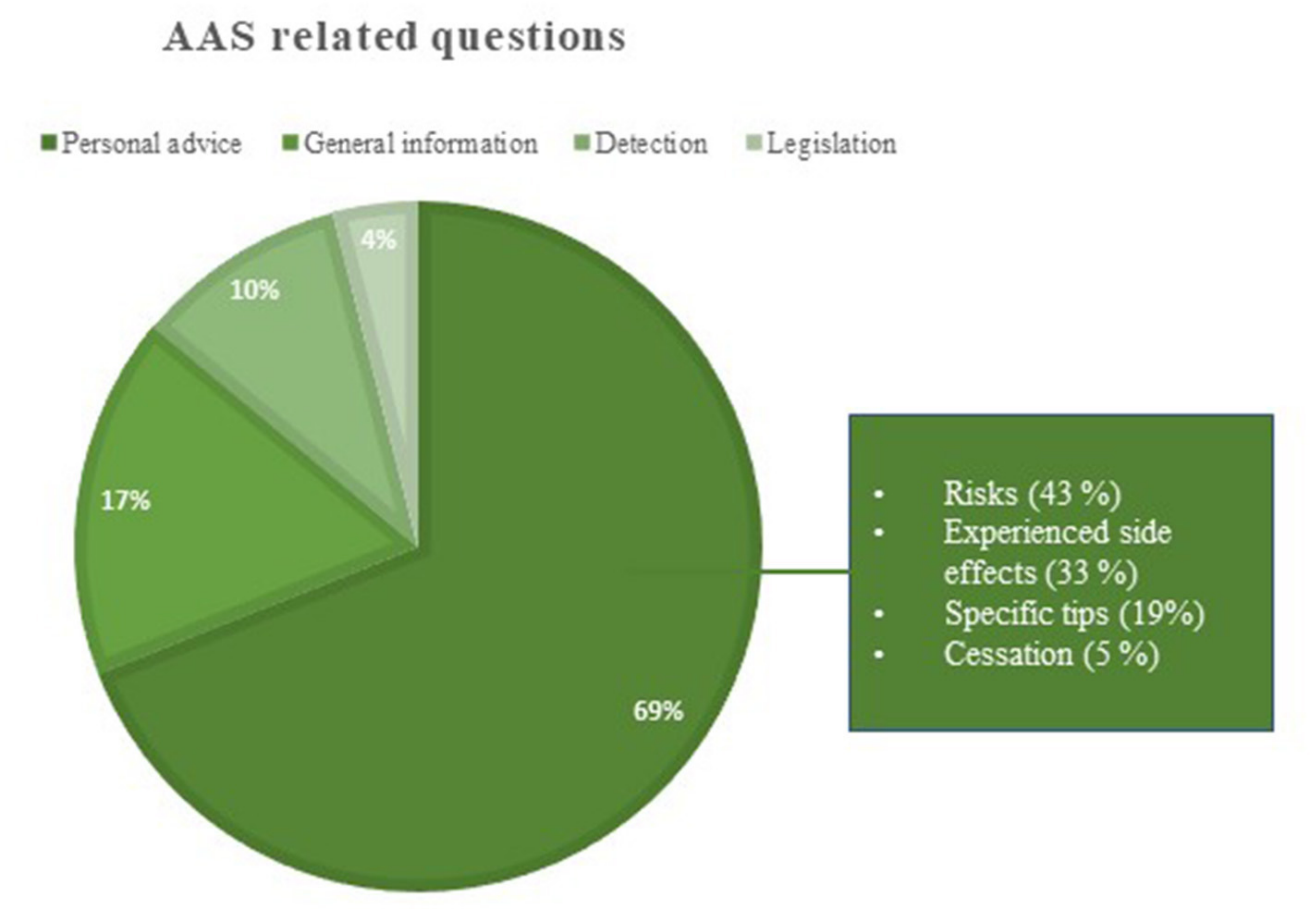
The Anabolic Androgenic Steroids (AAS) related questions were divided into four groups. The textbox shows what type of advice the questioner requested.

The AAS related questions (*n* = 2,924) were further divided into four groups based on the type of enquiry: personal advice, general information, detection, or legislation (Figure 2).

#### Personal Advice

**Figure 3:**
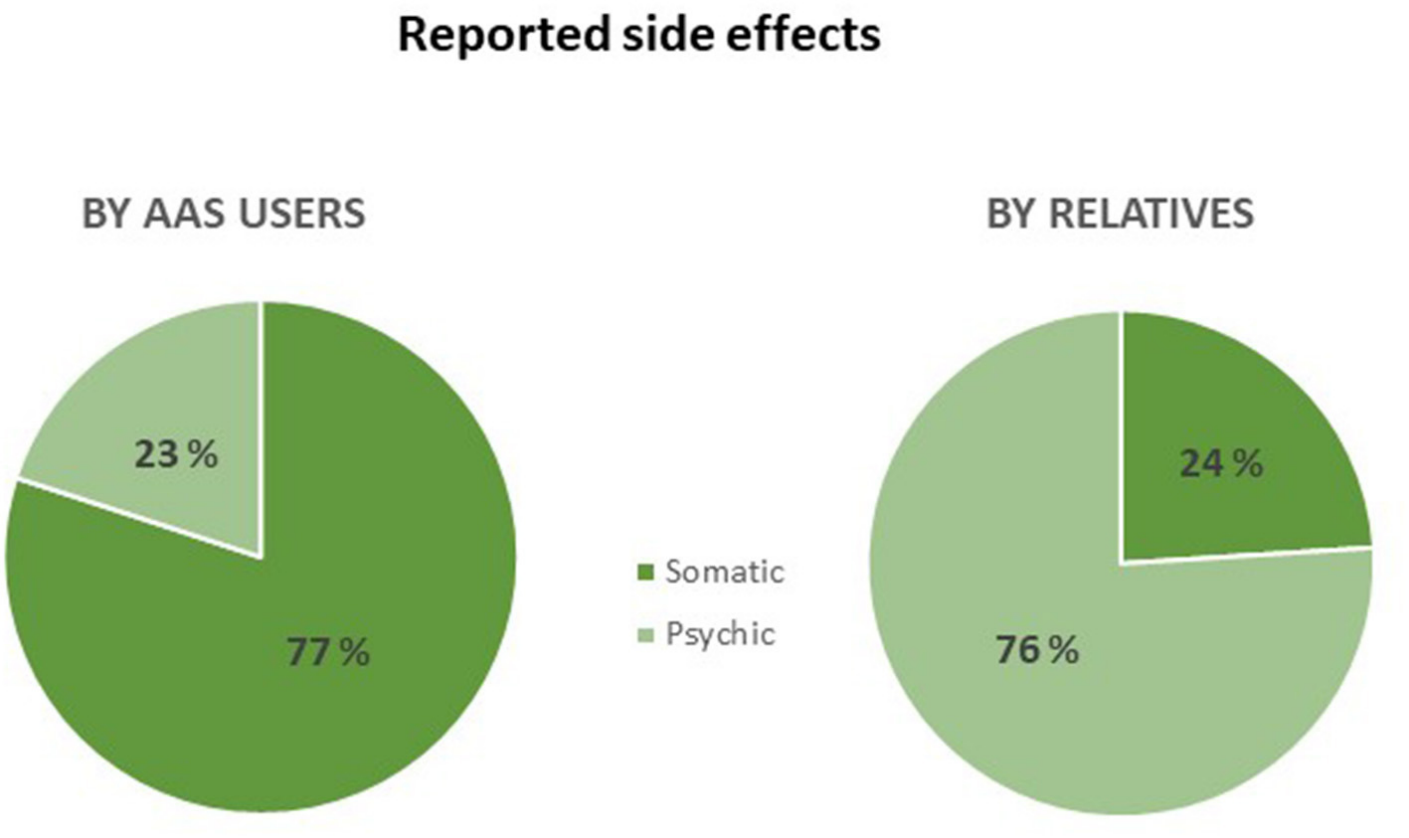
Percentage of psychic and somatic side effects reported by next of kin and abusers.

The largest group of AAS related questions concerned counseling, the questioners were help-seeking or asking for personal advice. The questions categorized as “advice” (*N* = 2,033) were further sub-analyzed to see what kind of advice that was requested (Figure 2). Most of the questioners expressed a concern about the risks, they had taken by using or having used AAS, or what kind of side-effects they might expect. Moreover, the AAS abusers were worried about symptoms and side effects they in fact had experienced when using AAS. AAS abusers expressed greater concerns about side effects of a physical character compared to relatives who were mainly concerned about psychic side effects (see Table 2; Figure 3). Symptoms of anxiety was the only side effect among the top five reported side effect by both AAS abusers and relatives. The most common questions from male AAS abusers were about gynecomastia, problems after injection of AAS, potency and reproductive issues. The questions about the sexual side effects were about decreased libido (sex drive), impotence, shrinkage, and pain in the testicles. The questioners were worried that these side effects would persist and not return to normal after stopping their use of AAS. Women reported menstrual disorders, clitoral enlargement, and voice change.

**Table 2:** The top five side effects experienced/described by next of kin and by anabolic androgenic steroids (AAS) abusers.

**Enquiries about experienced side effects**			
**Next of kin**	**Number**	**Abusers**	**Number**
Mood swings	59 (19%)	Gynecomastia	95 (13.8%)
Aggressivity	47 (15%)	Problems after injection of AAS	70 (10.2%)
Depression	20 (6.5%)	Anxiety	54 (8%)
Changes in personality	18 (5.8%)	Problems with the testicles	54 (8%)
Anxiety	16 (5.2%)	Potency problems	54 (8%)

Of all AAS related questions from relatives, 95% included a request for personal advice. Most commonly the questions were from a worried parent or a partner who sought advice on how to deal with the AAS abuser's perceived side effects and/or their behavior. The most commonly described physical side effects in men were sexual problems, acne and sleeping disorders. The questions regarding behavior were about the AAS user's personality having changed, i.e., the person was perceived as absent-minded, antisocial or with decreased empathy. In addition, they also described mood changes, aggression and the AAS abuser being frightening. When it comes to side effects in AAS using women, acne, menstrual disorders, clitoral enlargement and sweating was described.

Thirdly, the questioners often wanted specific tips about various AAS and co-used doping substances. Here, the AAS abusers searched for advice on choosing the right combination of AAS substances and dosage to minimize the androgen effect,- or wanted instructions on how to inject AAS correctly and/or information on which drugs to use to avoid side effects.

Finally, the questioners also requested advise on how to quit their use of AAS in the best possible way, i.e., without having to experience side effects. A common question was how they could get professional help for their side effects or for ending their AAS use.

#### General Information

Seventeen percent of the AAS related questions concerned general information about AAS, e.g., what happens in the body when using AAS, how AAS work and general questions about side effects (Figure 2). In this group it was not possible to determine who the questioner was.

#### Detection

Ten percent of the AAS related questions were about detection time and testing of AAS (Figure 2). These questions were mostly from AAS abusers. The questioners wanted to know if AAS were possible to detect in a routine drug test and for how long a certain AAS substance could be detected in the test. A few questions about detection, mostly about detection times came from professionals, such as social services, the police, prison, staff, customs, or health care staff.

#### Legislation

Only four percent of the AAS related questions concerned the Swedish doping law (Figure 2). Most were about the legal consequences of testing positive on AAS. The AAS abusers had questions about what the doping law entails and what penalties one can get for using AAS.

### AAS Substances

There were 16 different AAS substances that were specifically declared in the enquiries. The 10 most common AAS 2005–2018 and the most commonly stated substances per year are shown in Table 3.

**Table 3:** (a) The 10 most commonly specified anabolic androgenic steroids (AAS) during 2005–2018 and (b) the most popular substances per year.

**(a) The 10 most commonly asked substances in number (%) 2005–2018**					
	**Substance**	**Number (%)**		**Substance**	**Number (%)**
***1***	Testosterone	687 (35.0)	**6**	Oxandrolone	78 (3.9)
***2***	Methandienone	459 (23.0)	**7**	Oxymetholone	62 (3.1)
***3***	Nandrolon	273 (13.8)	**8**	Drostanolone	34 (1.7)
***4***	Stanozolol	180 (9.1)	**9**	Boldenone	28 (1.4)
***5***	Trenbolone	107 (5.3)	**10**	Methenolone	25 (1.2)
**(b) Most popular substance each year**					
2005					Methandienone
2006					Nandrolone
2007					Methandienone
2008					Testosterone
2009–2010					Methandienone
2011–2018					Testosterone

Testosterone and methandienone were the compounds with most enquiries, 35 and 23% of the total, respectively. The number of substance specific questions correlated with the rate of positive findings detected at our Drug Abuse Laboratory during the time-period 2015–2018, a total of 8,616 samples analyzed and 1,357 of these being identified as positive (8). The prevalence of the positive findings per substance correlated with the frequency of specific enquiries of the same substance (*R* = 0.58, *p* = 0.047).

### Other Doping Substances

In addition to AAS, questions were raised about growth hormone (GH), ephedrine, clenbuterol, selective androgen receptor modulators (SARM) and other doping classified agents and performing enhancing drugs (PED), i.e., peptides. Most questions were related to ephedrine (4.8%), whereas the frequencies of questions regarding other doping agents i.e., GH, peptides, clenbuterol, or prohormones were low (≤ 1.5% each).

**Figure 4:**
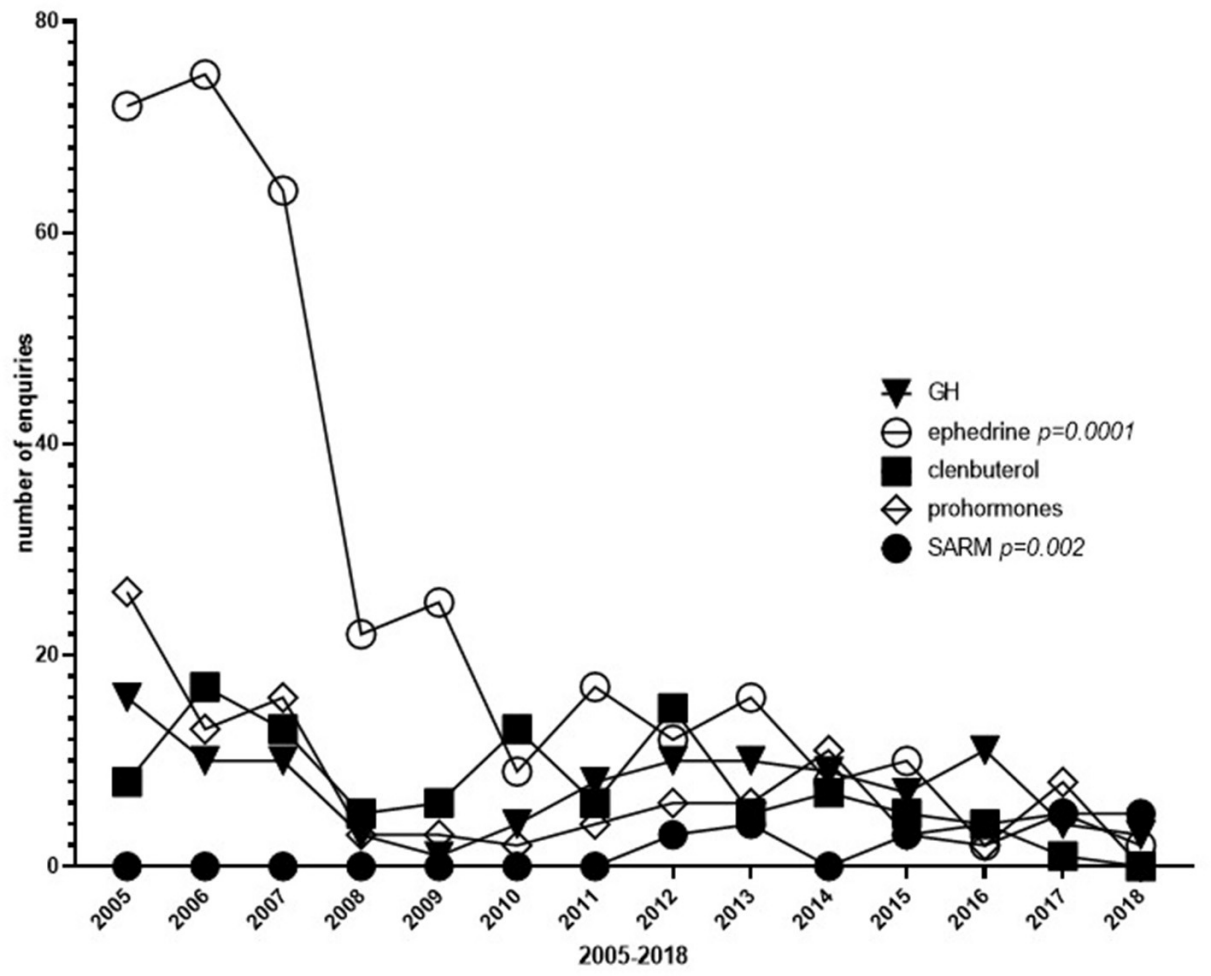
Frequency of questions regarding doping agents other than Anabolic Androgenic Steroids (AAS) between 2005 and 2018. GH, Growth Hormone; SARM, Selective Androgen Receptor Modulator.

The enquiries regarding ephedrine decreased from over 60 during 2005–2007 to below 20 after 2009, Figure 4. For SARMS an increase in the number of enquiries was noted, i.e., there were no enquiries regarding SARMs between 2005 and 2011, whereas from 2012 and onwards, in average 4 enquiries were submitted each year (Figure 4). The numbers of questions regarding other doping agents were fairly stable over the years (Figure 4).

## Discussion

This is to our knowledge the largest published report describing doping-related questions posed to an open, society-oriented doping information service. Most questions were formulated as general questions about the risks associated with AAS use rather than describing personally experienced side effects. It is possible that some subjects are not willing to admit their AAS use since AAS are illegal in Sweden, and/or due to fear of being judged, even though the questions were submitted anonymously. It is also possible that the questioners seeked information prior to planned abuse which would be in line with studies showing that AAS abusers seek for knowledge before making strategic choices e.g., to minimize harm and maximize effects (9).

It was not surprising that there were very few questions submitted by women as AAS abuse is mainly a male problem in the society. Nevertheless, even though women are more prone to the androgenic effects of AAS, i.e., they encounter side effects at much lower doses than men (10), some are still willing to expose themselves to these risks (11). Consequently, it is vital to provide information and support directed specifically to women as very little information is available.

Seventeen percent (with a variation during the years between 10 and 24%) of all AAS related enquiries came from persons with a relationship to an AAS abuser. These numbers corroborate with phone calls reported by the ADHL for the period 1993–2000 (7) and by a recent report from Anti-Doping Denmark including telephone calls and online submissions during 2006–2007 (6). It was evident that relatives and spouses expressed concerns about other side effects than the AAS abusers. Both next of kin and AAS abusers described anxiety in some form. The anxiety experienced by next of kin was related to an unsustainable family situation or to the consequences of the AAS abuser not wanting to or having problems with ending their AAS use. The anxiety of the AAS abusers, on the other hand, was about the risks they took by using AAS or that the side effects would be permanent and/or serious.

Relatives have previously reported behavioral changes, depression, decreased empathy, aggression and anxiety in AAS abusers (12). They also have questions about what kind of professional help they may expect and how they should act when a close family member uses AAS (7). Next of kin consider information about AAS important to be able to understand the side effects of AAS use (12). The need for support for the next of kin has been consistent over the years. Unfortunately, this is a neglected group, and no research is available describing their situation. It has been suggested that “co-dependence” (normalization of AAS behaviors), like for other drugs of abuse, may occur (13). In-depth interviews may be a way to access the existential dimension of next of kin experiences of living close to a person using AAS. By deeper knowledge, better tools could be developed for meeting and supporting relatives especially in a healthcare environment.

Testosterone and methandienone were the steroids most often mentioned in the online enquiries. These two substances were the two most abused AAS also in 1993–2000 according to telephone calls to the ADHL (7). Testosterone is the most common AAS detected, both in urine samples from AAS abusers (14) and in elite athletes (WADA) (15). Methandienone is also often detected in samples from AAS abusers, but not to the same extent (14, 16). The specific compounds used may in part be related to the availability in the society. Testosterone and methandienone accounted for 48 and 10% of all AAS confiscated in Sweden 2016–2018 (8), and 25 and 6.6% of all confiscated doping agents in Switzerland 2013–2014 (17).

An interesting observation herein was that the substance specific enquiries on our homepage reflected the analytes detected in healthcare urine samples i.e., samples often taken in addiction care units when AAS abuse is suspected. Monitoring of AAS enquiries together with customs seizure may serve as indicators on which AAS compounds to include in AAS testing outside elite sports. As opposite to elite sport testing where advanced and sensitive analytical methods are applied according to WADA guidelines, a simplified mass-spectrometry method focusing on the detection of the most common AAS in the society may facilitate more clinical laboratories to offer AAS testing (8). Analysis of AAS is diagnostically more reliable than use of clinical biomarkers (i.e., serum testosterone and gonadotropin levels) and subsequently an important tool to identify AAS abusers in order to offer them adequate treatment strategies (18).

SARMs were added to the WADA prohibited list in 2008, as a potential misuse was suspected based on the anabolic properties seen in clinical studies (19, 20). Subsequently, all samples obtained from elite athletes are subject to testing of several SARMs in WADA accredited laboratories and atypical analytical findings of SARMs have increased throughout the years (20). Regrettably, SARMs are not regularly analyzed in forensic and healthcare samples as they are not per definition steroids, so the prevalence of their use in the society is not known. Our results indicate that the use of SARMs may increase also outside the arena of elite sports. There are anecdotal statements from individuals contacting our ADHL that these substances are “new anabolic preparations with improved anabolic effects and less adverse side effects.” This perception of SARMs is worrisome, as SARMs have in fact been associated with severe toxicity (21).

Notably, 26% of all enquiries concerned dietary supplements. Dietary supplements include protein powder, creatine, vitamins, herbals etc., and are taken for muscle building, to increase energy, stay healthy and for weigh loss purposes. Dietary supplement use is common among athletes at all levels, mainly due to the easy availability *via* internet and assertive marketing procedures (6, 22). Even though dietary supplements are not prohibited or illegal, there are concerns of their safety, particularly since AAS have been repeatedly detected in dietary supplements (23, 24). Therefore, to avoid inadvertent doping there is a general recommendation for elite athletes not to use supplements as even trace amounts of AAS can be detected in urine (25). In addition, some dietary supplements (particularly “fat burning” dietary supplements), may be deliberately adulterated with e.g., β-agonists, erythropoiesis stimulating agents and stimulants which may constitute a health concern (26).

The enquiries regarding ephedrine have decreased over the years. Whether this reflects a reduced use in the society is not known. Studies indicate that ephedrine and similar drugs are popular not only in sports but as “brain-doping” among adolescents (27) and the prescription rate has increased drastically in parallel with the increase in the diagnoses of attention deficit hyperactivity disorder (ADHD) (28, 29). It is possible that many AAS abusers now uses ADHD medicines therapeutically rather than obtaining them from the black market, resulting in less online concerns being submitted.

This study has limitations related to the anonymous data collection. For example, several questions may come from the same individual and the difference between calls per year do not necessary reflect the numbers of people concerned. The numbers of enquiries may also be distorted due to shorter periods of technical problems on the ADHL homepage. Moreover, the categorization of the enquiries by the ADHL staff was subjective. Nevertheless, this anthology includes the largest internet-based material of AAS related enquiries and provides an understanding on what information AAS abusers are seeking.

## Conclusion

This compilation indicates that there is a continuous need for persons outside elite sport to access information and support in anti-doping related questions, particularly regarding concerns and risks associated with AAS use. Even though more information has become available throughout the years in diverse internet webpages and forums, there is still need for professional advice, both for AAS abusers themselves and for persons in a close relationship to an AAS abuser. This highlights the importance of providing personalized medical, nursing and social support to AAS abusers and their next of kin. It is also important to continue to spread information and educate about AAS in the society, i.e., for healthcare givers and in education programs.

## Data Availability Statement

The original contributions presented in the study are included in the article/supplementary material, further inquiries can be directed to the corresponding author.

## Author Contributions

SB gathered the online enquiries. All the authors analyzed the data, read, and approved the final manuscript.

## Conflict of Interest

The authors declare that the research was conducted in the absence of any commercial or financial relationships that could be construed as a potential conflict of interest.

## Publisher's Note

All claims expressed in this article are solely those of the authors and do not necessarily represent those of their affiliated organizations, or those of the publisher, the editors and the reviewers. Any product that may be evaluated in this article, or claim that may be made by its manufacturer, is not guaranteed or endorsed by the publisher.

## References

[1] Bhasin S Woodhouse L Storer TW. Proof of the effect of testosterone on skeletal muscle. J Endocrinol. (2001) 170:27–38. 10.1677/joe.0.1700027 11431134

[2] Evans NA. Gym and tonic: a profile of 100 male steroid users. Br J Sports Med. (1997) 31:54–8. 10.1136/bjsm.31.1.54 9132214 PMC1332477

[3] Parkinson AB Evans NA. Anabolic androgenic steroids: a survey of 500 users. Med Sci Sports Exerc. (2006) 38:644–51. 10.1249/01.mss.0000210194.56834.5d 16679978

[4] Kanayama G Brower KJ Wood RI Hudson JI Pope HG Jr. Anabolic-androgenic steroid dependence: an emerging disorder. Addiction (Abingdon, England). (2009) 104:1966–78. 10.1111/j.1360-0443.2009.02734.x 19922565 PMC2780436

[5] Kaufman MJ Kanayama G Hudson JI Pope HG Jr. Supraphysiologic-dose anabolic-androgenic steroid use: a risk factor for dementia? Neurosci Biobehav Rev. (2019) 100:180–207. 10.1016/j.neubiorev.2019.02.014 30817935 PMC6451684

[6] Bojsen-Møller J Christiansen AV. Use of performance- and image-enhancing substances among recreational athletes: a quantitative analysis of inquiries submitted to the Danish anti-doping authorities. Scand J Med Sci Sports. (2010) 20:861–7. 10.1111/j.1600-0838.2009.01023.x 19843266

[7] Eklof AC Thurelius AM Garle M Rane A Sjoqvist F. The anti-doping hot-line, a means to capture the abuse of doping agents in the Swedish society and a new service function in clinical pharmacology. Eur J Clin Pharmacol. (2003) 59:571–7. 10.1007/s00228-003-0633-z 13680032

[8] Widing E Beck O Andersson A Bragd J Hansson T. Increased availability needed for analysis of anabolic androgenic steroids outside of sports - proposal of a uniform clinical analysis method in Sweden. Lakartidningen. (2021) 118:20047. 33502750

[9] Cohen J Collins R Darkes J Gwartney D. A league of their own: demographics, motivations and patterns of use of 1,955 male adult non-medical anabolic steroid users in the United States. J Int Soc Sports Nutr. (2007) 4:12. 10.1186/1550-2783-4-12 17931410 PMC2131752

[10] Börjesson A Gårevik N Dahl ML Rane A Ekström L. Recruitment to doping and help-seeking behavior of eight female AAS users. Subst Abuse Treat Prev Policy. (2016) 11:11. 10.1186/s13011-016-0056-3 26945991 PMC4779574

[11] Börjesson A Möller C Hagelin A Vicente V Rane A Lehtihet M. Male anabolic androgenic steroid users with personality disorders report more aggressive feelings, suicidal thoughts, and criminality. Medicina (Kaunas, Lithuania). (2020) 56:265. 10.3390/medicina56060265 32481676 PMC7353874

[12] Havnes IA Jørstad ML Wisløff C. Anabolic-androgenic steroid users receiving health-related information; health problems, motivations to quit and treatment desires. Subst Abuse Treat Prev Policy. (2019) 14:20. 10.1186/s13011-019-0206-5 31096999 PMC6524231

[13] Region Örebro Län. Dopning - översikt, vård och behandling samt idéskiss till ett nationellt kompetenscentrum, slutrapport från Nationellt kompetensutvecklingsprojekt fÖr dopningsproblematik. (2016) Region Örebro Län.

[14] Börjesson A Lehtihet M Andersson A Dahl ML Vicente V Ericsson M. Studies of athlete biological passport biomarkers and clinical parameters in male and female users of anabolic androgenic steroids and other doping agents. Drug Test Anal. (2020) 12:514–23. 10.1002/dta.2763 31925932

[15] Agency WA-D,. Doping Testing Figures. (2019). Available online at: https://www.wada-ama.org/sites/default/files/resources/files/2019_anti-doping_testing_figures_en.pdf (accessed November 15, 2021).

[16] Lood Y Eklund A Garle M Ahlner J. Anabolic androgenic steroids in police cases in Sweden 1999-2009. Forensic Sci Int. (2012) 219:199–204. 10.1016/j.forsciint.2012.01.004 22269132

[17] Weber C Krug O Kamber M Thevis M. Qualitative and semiquantitative analysis of doping products seized at the Swiss border. Subst Use Misuse. (2017) 52:742–53. 10.1080/10826084.2016.1263665 28156209

[18] Anawalt BD. Detection of anabolic androgenic steroid use by elite athletes and by members of the general public. Mol Cell Endocrinol. (2018) 464:21–7. 10.1016/j.mce.2017.09.027 28943276

[19] Dalton JT Taylor RP Mohler ML Steiner MS. Selective androgen receptor modulators for the prevention and treatment of muscle wasting associated with cancer. Curr Opin Support Palliat Care. (2013) 7:345–51. 10.1097/SPC.0000000000000015 24189892

[20] Thevis M Schanzer W. Detection of SARMs in doping control analysis. Mol Cell Endocrinol. (2018) 464:34–45. 10.1016/j.mce.2017.01.040 28137616

[21] Fonseca G Dworatzek E Ebner N Von Haehling S. Selective androgen receptor modulators (SARMs) as pharmacological treatment for muscle wasting in ongoing clinical trials. Expert Opin Investig Drugs. (2020) 29:881–91. 10.1080/13543784.2020.1777275 32476495

[22] Garthe I Maughan RJ. Athletes and supplements: prevalence and perspectives. Int J Sport Nutr Exerc Metab. (2018) 28:126–38. 10.1123/ijsnem.2017-0429 29580114

[23] De Cock KJ Delbeke FT Van Eenoo P Desmet N Roels K De Backer P. Detection and determination of anabolic steroids in nutritional supplements. J Pharm Biomed Anal. (2001) 25:843–52. 10.1016/S0731-7085(01)00396-X 11377067

[24] Geyer H Parr MK Mareck U Reinhart U Schrader Y Schanzer W. Analysis of non-hormonal nutritional supplements for anabolic-androgenic steroids - results of an international study. Int J Sports Med. (2004) 25:124–9. 10.1055/s-2004-819955 14986195

[25] Micalizzi G Huszti K Palinkas Z Mandolfino F Martos E Dugo P. Reliable identification and quantification of anabolic androgenic steroids in dietary supplements by using gas chromatography coupled to triple quadrupole mass spectrometry. Drug Test Anal. (2021) 13:128–39. 10.1002/dta.2929 32959986

[26] Collins J Maughan RJ Gleeson M Bilsborough J Jeukendrup A Morton JP. UEFA expert group statement on nutrition in elite football. Current evidence to inform practical recommendations and guide future research. Br J Sports Med. (2021) 55:416. 10.1136/bjsports-2019-101961 33097528

[27] Lakhan SE Kirchgessner A. Prescription stimulants in individuals with and without attention deficit hyperactivity disorder: misuse, cognitive impact, and adverse effects. Brain Behav. (2012) 2:661–77. 10.1002/brb3.78 23139911 PMC3489818

[28] Giacobini M Medin E Ahnemark E Russo LJ Carlqvist P. Prevalence, patient characteristics, and pharmacological treatment of children, adolescents, and adults diagnosed with ADHD in Sweden. J Atten Disord. (2018) 22:3–13. 10.1177/1087054714554617 25376193

[29] Polyzoi M Ahnemark E Medin E Ginsberg Y. Estimated prevalence and incidence of diagnosed ADHD and health care utilization in adults in Sweden - a longitudinal population-based register study. Neuropsychiatr Dis Treat. (2018) 14:1149–61. 10.2147/NDT.S155838 29765219 PMC5944447

